# The complete mitochondrial genome and phylogenetic analysis of the Jawala paste shrimp, *Acetes indicus* (Decapoda, Dendrobranchiata, Sergestidae)

**DOI:** 10.1080/23802359.2026.2722802

**Published:** 2026-09-29

**Authors:** Ju Eun Cha, Hyeon Ju Na, Seongdo Lee, Sang Jo Kim, Jang Wook Lee, Jehee Lee, Seong Don Hwang

**Affiliations:** a Department of Convergence Interdisciplinary Education of Maritime & Ocean Logistics, Korea Maritime & Ocean University, Busan, Republic of Korea; b Ocean Science and Technology School, Korea Maritime & Ocean University, Busan, Republic of Korea; c General Affairs Division, National Fishery Products Quality Management Service, Busan, Republic of Korea; d Department of Marine Life Sciences & Center for Genomic Selection in Korean Aquaculture, Jeju National University, Jeju, Republic of Korea; e Division of Convergence on Marine Science, Korea Maritime & Ocean University, Busan, Republic of Korea

**Keywords:** *Acetes indicus*, Jawala paste shrimp, Mitogenome, phylogenetic tree

## Abstract

We have analyzed the complete mitochondrial genome sequence of the Jawala paste shrimp, *Acetes indicus*. The genome sequence was 15,785 bp in length and encompassed 39 features: 13 protein-coding genes (PCGs), 23 transfer RNAs, 2 ribosomal RNAs, and a control region (CR). Although typical metazoan mitochondrial genomes contain 22 tRNAs, *A. indicus* has additional tRNA gene due to a tandem duplication of the trnS1 gene, resulting in trnS1(AGA) and trnS1(AGC), like *Acetes chinensis*. Percent identity and phylogenetic tree indicated that *A. indicus* was closely related to *A. chinensis* with a high sequence similarity of 86.2%. The complete mitochondrial genome of *A. indicus* may provide useful genetic resources for the genus *Acetes* and is expected to facilitate comprehensive phylogenetic and evolutionary investigations within the family Sergestidae.

## Introduction

Salted and fermented seafood played a significant role in regard to traditional food culture, particularly in regions with hot and humid climates (Peralta et al. 2008; Koral et al. 2013). In Korea, traditional salted fermented products are prepared from fish, shellfish, and shrimp (Park et al. 2023). Among the various types, salted opossum shrimps are commonly used as flavoring agents in a wide range of traditional Korean dishes.

Salted opossum shrimp consists of the species, *Acetes* in Korea (Guan et al. 2011; Park and Kim 2022). However, they are less than approximately 2–3 cm in size, making it difficult to identify the species morphologically (Park et al. 2021). *A. indicus*, the type species of the genus *Acetes*, was first described by H. Milne Edwards in 1830 based on specimens collected from the Ganges Delta. This species is known to inhabit the coastal regions of the Indian Ocean, where it occurs primarily in surface to intermediate waters, and exhibits planktonic behavior typical of the genus (Vereshchaka et al. 2016). Genetic information on *A. indicus* still remains limited, even though it is commercially important species.

This study aimed to determine the complete mitochondrial genome of *A. indicus* to provide foundational genomic data to support species and taxonomic identification within the genus *Acetes*. In addition, phylogenetic analysis of related families was performed in order to clarify the evolutionary relationships within the genus.

## Materials and methods

### Experimental samples

Salted opossum shrimp samples that imported into Korea from Malaysia were desalted by immersing them in distilled water twice, for 3 h each and homogenized. DNA was extracted using the QIAamp DNA Mini Kit (QIAGEN, Germany). To identify species of samples, conventional PCR was conducted to amplify the cytochrome c oxidase subunit I (COI) gene with specific primers by Park et al. (2021). Amplification was conducted ExPremier^™^ DNA Polymerase (TaKaRa, Japan) 10 µL, reaction water 7 µL, forward and reverse primers 1 µL for each, DNA template 1 µL. The quality and concentration were verified using 1.5% agarose gel electrophoresis, purified using Expin^™^ PCR SV (GeneAll, Korea). PCR products were sequenced by Bionics (Korea), using an ABI 3730xl DNA Analyzer platform (Applied Biosystems, USA). Obtained sequence was analyzed using the Nucleotide BLAST (https://blast.ncbi.nlm.nih.gov/Blast.cgi), and it exhibited 99.82 ∼ 100% identity with the previously reported *A. indicus* type I (Park et al. 2021) COI gene. The sample was stored at the Korea Maritime and Ocean University, Busan, Republic of Korea (Figure 1, Fiq24600083, Contact: Assoc. Prof. Seong Don Hwang, sdhwang@kmou.ac.kr).

**Figure 1.**
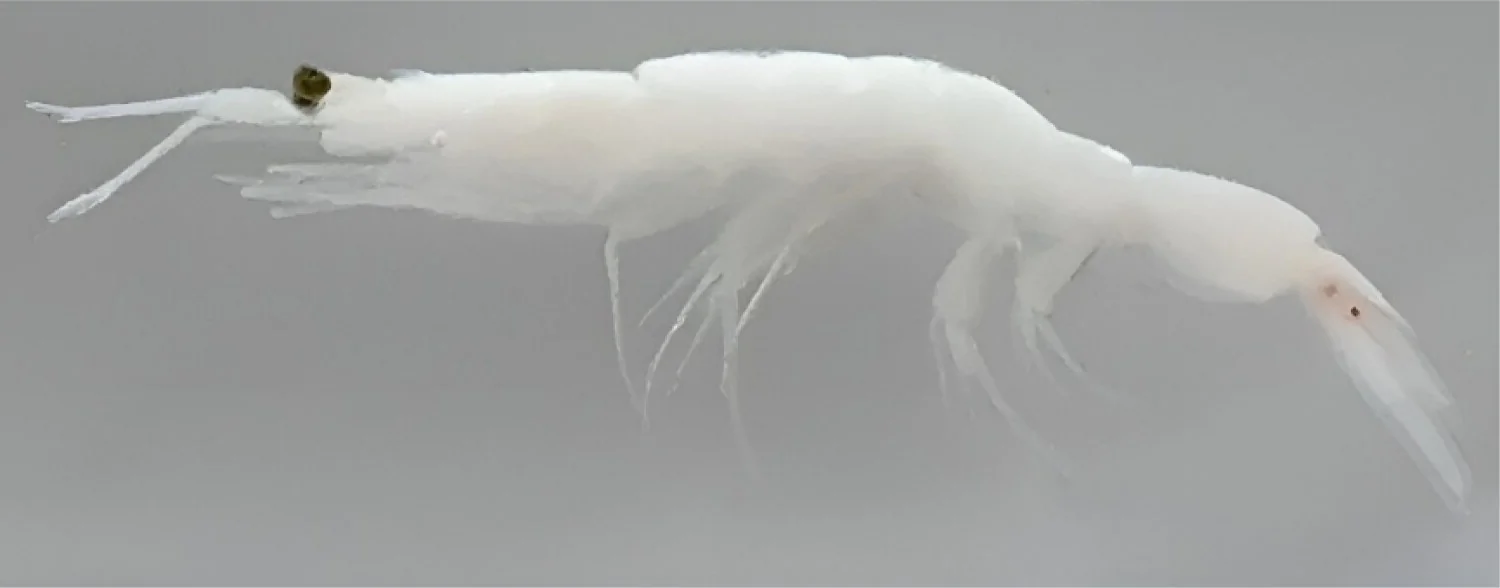
The lateral view of the *Acetes indicus* (H. Milne Edwards, 1830). Pictures courtesy of Ju Eun Cha.

### Cloning

Previously reported primer sets were used to obtain fragments of mitochondrial genome, and additional primers were newly designed to fill the gaps between the fragments, resulting in the complete mitochondrial genome sequence (Table S1 and Figure S1). Purified amplicons were cloned by TOPcloner^™^ TA kit (Enzynomics, Korea) according to manufacturer’s instructions, and the nucleotide sequence were determined by Bionics (Korea). Sequences were compared with other Decapoda species published on NCBI GenBank database for verification.

### Sequence annotation and phylogenetic analysis

The mitogenome was annotated by BioEdit (v7.7.1) and MEGA (v11.0.10). We identified the initiation and termination codons by aligning our data against the *A. chinensis* (GenBank NC_017600.1). Open reading frames were predicted by the ORF finder (https://www.ncbi.nlm.nih.gov/orffinder/). The circular map of the genome and GC content/skew were rendered with the Proksee (https://proksee.ca/).

To determine evolutionary relationships, a Maximum Likehood (ML) phylogenetic tree was constructed based on 18 complete mitochondrial genomes (15,035 positions). The ML analysis was performed in MEGA using the Tamura-Nei substitution model, selected *via* the BIC criterion. Branch was statistically evaluated with 1,000 bootstrap iterations.

## Results

### Mitochondrial DNA analysis

The mitochondrial genome of *A. indicus* (GenBank PQ849921.1) was 15,785 bp in length and included 39 features: 13 PCGs (COI, COII, ATP8, ATP6, COIII, ND3, ND5, ND4, ND4L, ND6, CYTB, ND1, ND2), 23 tRNAs (trnL2, trnK, trnD, trnG, trnA, trnR, trnN, trnS1^AGA^, trnS1^AGC^, trnE, trnF, trnH, trnT, trnP, trnS2, trnL1, trnV, trnI, trnQ, trnM, trnW, trnC, trnY), 2 rRNAs (lrRNA, srRNA), and a CR (Figure 2A). Although typical metazoan mitochondrial genomes contain 22 tRNAs, *A. indicus* has an additional tRNA gene, trnS1^AGA^ and trnS1^AGC^ (Figure 2B). There are no cis/trans-splicing genes in the mitochondrial genome. All PCGs start with a standard ATN codon, except for COI, which starts with an ACG codon (Table S3). Moreover, the PCGs terminate with stop codons TAA and TAG, except for COII, COIII, ND3 end with a single T codon and represent an incomplete stop codon. The lengths of the tRNAs range from 65 bp to 71 bp, and the lrRNA and srRNA genes are 1,369 bp and 838 bp. The nucleotide composition is 37.2% A, 34.6% T, 16.9% C, 11.3% G, showing a high AT bias with an AT skew of 0.036 and a GC skew of −0.201 for the complete mitochondrial genome. The CR is 764 bp long, and it has an AT-rich aspect.

**Figure 2.**
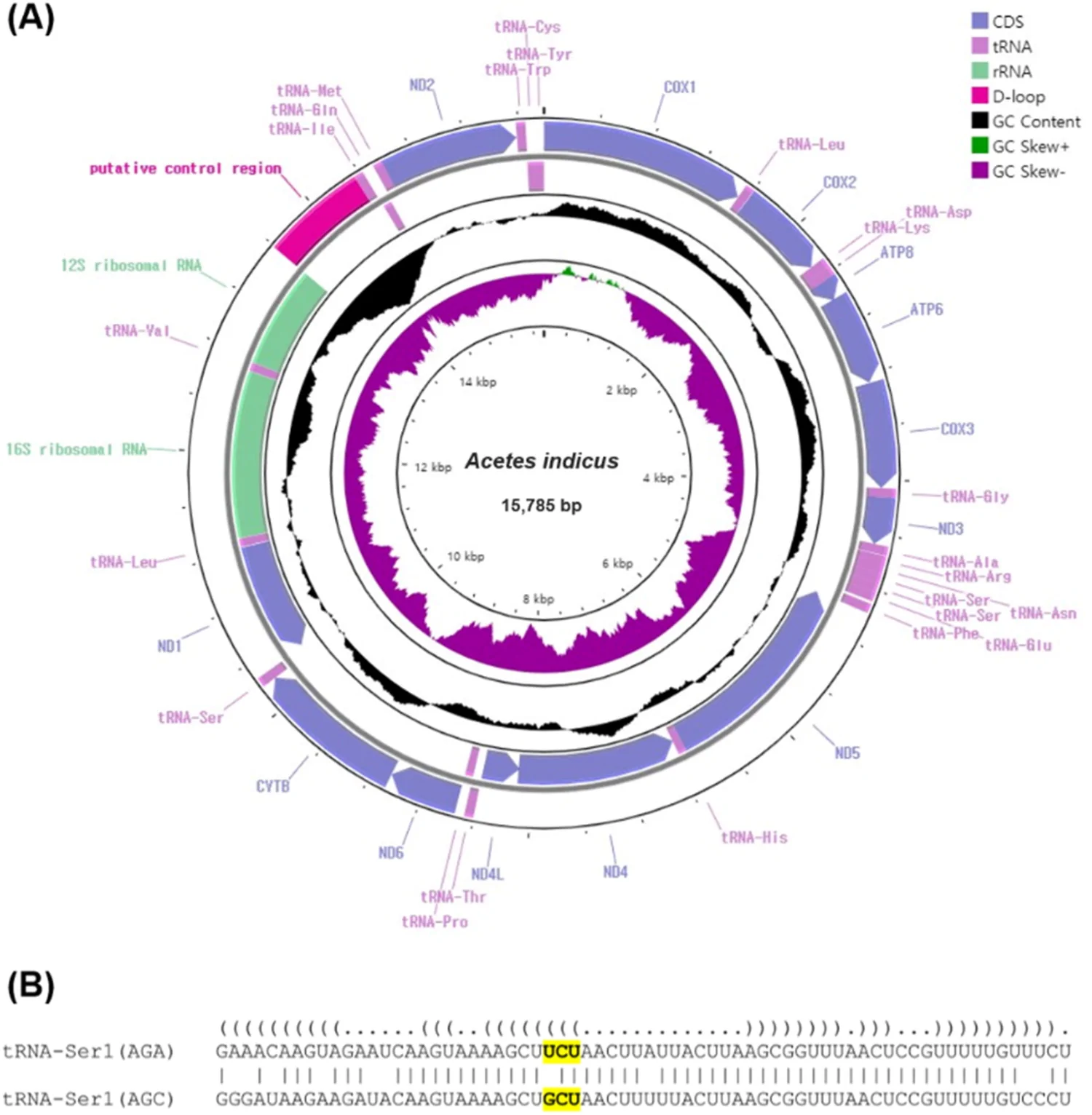
(A) The circular mitochondrial genome map of the Jawala paste shrimp (*Acetes indicus*). Except for the ND1, ND4L, ND4, and ND5 genes, all other coding sequences (CDS) are oriented in the clockwise direction. Both ribosomal RNAs (lrRNA and srRNA) are located on the L-strand. Similarly, most tRNAs are transcribed clockwise, aside from tRNA-His, tRNA-Pro, tRNA-Leu2, tRNA-Val, tRNA-Gln, tRNA-Cys, and tRNA-Tyr. The inner circles represent the GC content (black) and GC skew (green and purple) across the genome; (B) Alignment of the trnS1^AGA^ and trnS1^AGC^ sequences, highlighting the presence of the additional tRNA-Ser that results in 23 tRNAs in this species. The anticodon stem and loop structures are denoted by parentheses ‘(‘,’)’ and periods ‘.’, respectively. Vertical lines illustrate identical nucleotide matches, while the anticodon regions are distinctly highlighted.

### Phylogenetic analysis

Phylogenetic analysis was conducted using the previously reported complete mitochondrial genome sequences of *A. indicus* and six related families (Figure 3). Phylogenetic analysis showed that *A. indicus* clustered with the *A. chinensis* group of the family Sergestidae, forming a strongly supported clade (bootstrap value = 100) and high similarity of 86.2%. This close clustering suggested a high degree of genetic similarity between the two species. Other Sergestidae members, such as *Sergia lucens* and *Lucensosergia lucens*, were placed on separate branches, indicating a more distant relationship to the genus *Acetes*. The overall tree topology was consistent with established taxonomic classifications, with species from Penaeidae, Solenoceridae, Alvinocarididae, Palaemonidae, and Palinuridae family clearly forming distinct monophyletic clades.

**Figure 3.**
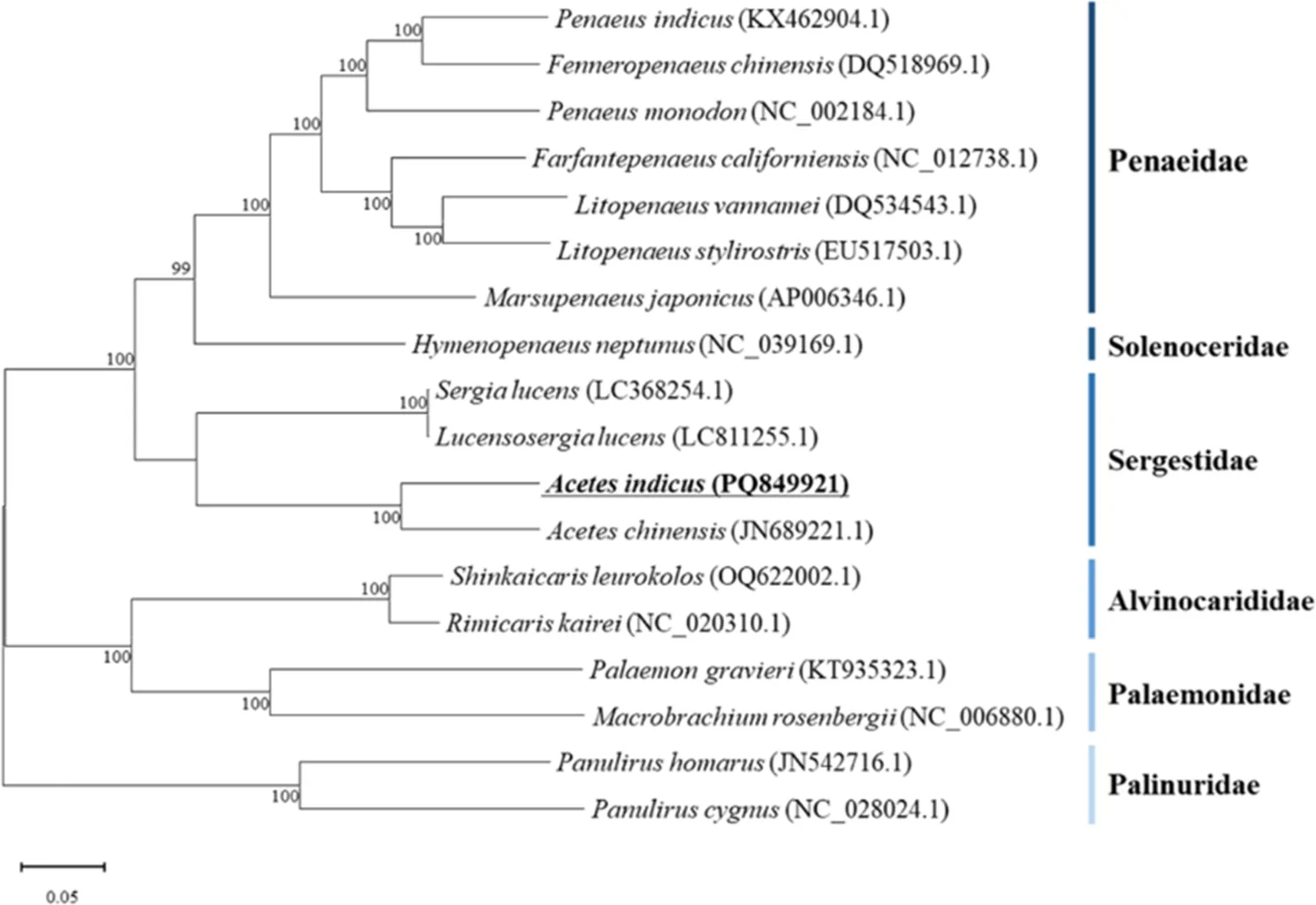
Maximum-likelihood phylogenetic tree based on the complete mitochondrial genome sequences of A. indicus and 17 other decapod species: KX462904.1 (Katneni et al. 2021), DQ518969.1 (Shen et al. 2007), NC_002184.1 (Wilson et al. 2000), NC_012738.1 (Gutiérrez-Millán et al. 2002), DQ534543.1 (Shen et al. 2007), EU517503.1 (Peregrino-Uriarte et al. 2009), AP006346.1 (Yamauchi et al. 2004), NC_039169.1 (Cheng et al. 2018), LC368254.1 (Kawato et al. 2018), LC811255.1 (Hirai et al. 2025), JN689221.1 (Kim et al. 2012), OQ622002.1 (Wang et al. 2023), NC_020310.1 (Yang et al. 2013), KT935323.1 (Kim et al. 2017), NC_006880.1 (Miller et al. 2005), JN542716.1 (Xiao et al. 2017), NC_028024.1 (Kim et al. 2016). The tree was inferred using the Tamura-Nei substitution model. Bootstrap support values (based on 1,000 replicates) are indicated at each node; accession numbers are indicated after the species names.

## Discussion and conclusion

This study reports the first complete mitochondrial genome of *A. indicus*, along with a phylogenetic analysis to infer its evolutionary position among related taxa. The overall structure and length of the *A. indicus* mitogenome are highly conserved and comparable to those of other decapod species.

*A. indicus* contains 23 tRNAs, deviating from the typical metazoan 22 tRNAs (Table S2) due to a tandem duplication of the trnS1 gene (trnS1^AGA^ and trnS1^AGC^). This feature, also reported in *A. chinensis* (Kim et al. 2012), supports their close phylogenetic relationship and shared evolutionary history. Furthermore, the incomplete stop codons observed in COII, COIII, and ND3 are typical features commonly formed in the order Decapoda, which are presumed to be completed post-transcriptional polyadenylation (Wilson et al. 2000; Shen et al. 2007).

A high AT content in CR (89.5%) is a typical feature of Decapoda (Kim et al. 2012; Wang et al. 2025). The distinct nucleotide composition biases, such as the high AT bias and negative GC skew reflect asymmetric mutational constraints heavily imposed on the mitochondrial strands during replication and transcription (Hassanin et al. 2005). The CR exhibits considerable variation in sequence length and composition among species. This reflects species-specific regulatory patterns. Its accurate characterization indicates the reliability and integrity of the mitogenome sequence (Kim et al. 2012; Sun et al. 2023).

Phylogenetic analysis showed that *A. indicus* clustered closely with *A. chinensis*, reflecting a high mitochondrial genome similarity. Until now, the complete mitochondrial DNA sequence within the genus *Acetes* had only been reported for *A. chinensis*. Therefore, this study provides genomic resources that are expected to facilitate future research on the taxonomy and evolution of the *Acetes* species.

## Supplementary Material

Supplemental material_final.docx

## Data Availability

The genome sequence data is available in GenBank of NCBI (https://www.ncbi.nlm.nih.gov/) under accession no. PQ849921.
