# Supplementary material for: The complete mitochondrial genome and phylogenetic analysis of the Jawala paste shrimp, Acetes indicus (Decapoda, Dendrobranchiata, Sergestidae)
Source: Mitochondrial DNA B Resour. 2026 Sep 29;11(10):1246–50. doi: 10.1080/23802359.2026.2722802 (PMC13629765; doi:10.1080/23802359.2026.2722802)
Supplement: Supplementary file 1 [file TMDN_A_2722802_SM5396.docx]

**The complete mitochondrial genome and phylogenetic analysis of the Jawala paste shrimp, *Acetes indicus* (Decapoda, Dendrobranchiata, Sergestidae)**

| **Table S1.** Primers used in this study. | | | | |
| --- | --- | --- | --- | --- |
| Primer | Sequences (5’-3’) | Reference | Gene position | |
|  |  |  | From | To |
| LCO1490 | GGTCAACAAATCATAAAGATATTGG | Folmer et al. (1994) | 42 | 693 |
| HCO2198 | TAAACTTCAGGGTGACCAAAAAATCA |  |  |  |
| HCO_2F | CCAGATATAGCTTTTCCTCGGATAAATAATATAAG | In this study | 298 | 2,220 |
| Mal_2R | TACTGATACACTTTCTACAACAATAGGGTTAAATC |  |  |  |
| 379F | GGAACAGGATGAACWGTT | Miller & Austin (2006) | 1,967 | 3,423 |
| 3423R | CTCGTCATCATTGAATTA |  |  |  |
| Mal_4F | GGTTTCGACTCCTAGATGTTGATAATC | In this study | 1,992 | 3,630 |
| 15_4R | CTGTATTGAGAAGAGGGATTTGGAAC |  |  |  |
| L4744-CO3 | THACWATAWWTCAATGATGACGAGA | Yamauchi et al. (2004) | 3,451 | 5,194 |
| H6375-ND5 | TTTDTWGGDTCWATGTGGTTTWTACC |  |  |  |
| 15N_1F | CCAACCATTATCTCCTACTCTCTGAAAATG | In this study | 5,114 | 6,834 |
| 24R_3 | CTGGCAAAGTTCGTGAGTATTTAGTTC |  |  |  |
| L7238-His | GCAGAGAAGGGWAKTTGAGCTCTTTT | Yamauchi et al. (2004) | 6,767 | 7,474 |
| H8481-ND5 | TAATGGTWKCTCACGGKWTCTGTTC |  |  |  |
| L8321-ND4 | ATATGDGCHACAGAWGAGTAAGC | Yamauchi et al. (2004) | 7,354 | 8,448 |
| H9302-Thr | AAGAGGTTSTTTTGWWTTTGGTTTACAAGACC |  |  |  |
| L9101-ND4L | CCHAGGGCHCCTTCGCAWGC | Yamauchi et al. (2004) | 8,203 | 9,496 |
| H10315-CYB | GTGATDACHGTDGCTCCTCA |  |  |  |
| Cytb L10061-CYB | GGATTWTTTTTAGCKATRCATTACAC | Yamauchi et al. (2002)  Machida et al. (2002) | 9,245 | 9,800 |
| H10699-CYB | GCAAATAGAAAATATCATTCWGGTTG |  |  |  |
| L10410-CYB | TATGRGGGGGSTTTGCTGTWGATAAT | Yamauchi et al. (2004) | 9,593 | 11,125 |
| H11892-ND1 | GGWTATATTCAGATTCGWAAGGGDCC |  |  |  |
| Cytb_1F1 | GTTACGTTCTCCCTTGAGGACA | In this study | 9,465 | 13,014 |
| 12S_1R | TCTGTAAACGATAATCCCCGCAA |  |  |  |
| 16s 1471 | CCTGTTTANCAAAAACAT | Crandall & Fitzpatrick (1996)  Viker et al. (2006) | 11,309 | 11,881 |
| 16s 1472 | AGATAGAAACCAACCTGG |  |  |  |
| Crust-12Sf | CAGCAKYCGCGGTTAKAC | Mondal & Mandal (2020) | 12,718 | 13,286 |
| Crust-12Sr | ACACCTACTWTGTTACGACTTATCTC |  |  |  |
| 12S-CO 1F | GCTGCACCTTGATCTAATATACTAAC | In this study | 12,908 | 464 |
| 12S-CO 1R | AACAGGTAAGGAAAGTAATAATAATAAAGCAGTA |  |  |  |
| 12S-CO 7F | CTCAAGTCCTACTCAAGCTG |  | 14,620 | 464 |
| 12S-CO 1R | AACAGGTAAGGAAAGTAATAATAATAAAGCAGTA |  |  |  |

| **Table S2.** Comparative analysis of mitochondrial genome features in *A. indicus* and related shrimp species. | | | | | | | |
| --- | --- | --- | --- | --- | --- | --- | --- |
|  | *Acetes*  *indicus* | *Acetes*  *chinensis* | *Litopenaeus vannamei* | *Palaemon gravieri* | *Fenneropenaeus chinensis* | *Penaeus*  *monodon* | *Marsupenaeus japonicus* |
| Accession number | PQ849921 | JN689221 | DQ534543 | KT935323 | DQ518969 | NC_002184.1 | AP006346 |
| Length (bp) | 15,785 | 15,740 | 15,989 | 15,740 | 16,004 | 15,984 | 15,968 |
| PCGs | 13 | 13 | 13 | 13 | 13 | 13 | 13 |
| tRNAs | 23 | 23 | 22 | 22 | 22 | 22 | 22 |
| rRNAs | 2 | 2 | 2 | 2 | 2 | 2 | 2 |
| CR | 1 | 1 | 1 | 1 | 1 | 1 | 1 |

**Figure S1**. Positions of the amplified mitochondrial DNA fragments in *A. indicus.* PCR fragments highlighted in gray were obtained using previously reported primers, while those highlighted in black were amplified using newly designed primers based on the sequences of the initial fragments.

| **Table S3.** Annotation of the *A. indicus* complete mitochondrial genomes. | | | | | | | |
| --- | --- | --- | --- | --- | --- | --- | --- |
| Feature | Position number | | Size (bp) | Codon | | Intergenic  nucleotides | Strand |
|  | From | To |  | Start | Stop |  |  |
| COI | 1 | 1536 | 1536 | ACG | TAA | 2 | + |
| tRNA-Leu2 | 1539 | 1603 | 65 |  |  | 4 | + |
| COII | 1608 | 2295 | 688 | ATG | T* | 0 | + |
| tRNA-Lys | 2296 | 2365 | 70 |  |  | 0 | + |
| tRNA-Asp | 2366 | 2434 | 69 |  |  | 0 | + |
| ATPase 8 | 2435 | 2593 | 159 | ATC | TAA | -7 | + |
| ATPase 6 | 2587 | 3261 | 675 | ATG | TAA | 5 | + |
| COIII | 3267 | 4056 | 790 | ATG | T* | 1 | + |
| tRNA-Gly | 4058 | 4124 | 67 |  |  | 0 | + |
| ND3 | 4125 | 4476 | 352 | ATC | T* | 0 | + |
| tRNA-Ala | 4477 | 4543 | 67 |  |  | 4 | + |
| tRNA-Arg | 4548 | 4612 | 65 |  |  | -1 | + |
| tRNA-Asn | 4612 | 4678 | 67 |  |  | 0 | + |
| tRNA-Ser1(AGA) | 4679 | 4746 | 68 |  |  | 0 | + |
| tRNA-Ser1(AGC) | 4747 | 4814 | 68 |  |  | 0 | + |
| tRNA-Glu | 4815 | 4883 | 69 |  |  | 20 | + |
| tRNA-Phe | 4904 | 4973 | 69 |  |  | 0 | + |
| ND5 | 4974 | 6704 | 1731 | ATG | TAG | 0 | - |
| tRNA-His | 6705 | 6770 | 66 |  |  | -1 | - |
| ND4 | 6770 | 8107 | 1338 | ATG | TAG | -7 | - |
| ND4L | 8101 | 8400 | 300 | ATG | TAA | 2 | - |
| tRNA-Thr | 8403 | 8470 | 68 |  |  | 0 | + |
| tRNA-Pro | 8471 | 8537 | 67 |  |  | 2 | - |
| ND6 | 8540 | 9070 | 531 | ATT | TAA | -1 | + |
| Cyt b | 9070 | 10206 | 1137 | ATG | TAG | -2 | + |
| tRNA-Ser2 | 10205 | 10275 | 71 |  |  | 18 | + |
| ND1 | 10294 | 11232 | 939 | ATA | TAG | 6 | - |
| tRNA-Leu1 | 11239 | 11307 | 69 |  |  | 3 | - |
| lrRNA | 11311 | 12679 | 1369 |  |  | 3 | - |
| tRNA-Val | 12683 | 12752 | 70 |  |  | 2 | - |
| srRNA | 12755 | 13592 | 838 |  |  | 0 | - |
| CR | 13593 | 14356 | 764 |  |  | 0 | + |
| tRNA-Ile | 14357 | 14425 | 69 |  |  | 4 | + |
| tRNA-Gln | 14430 | 14498 | 69 |  |  | 13 | - |
| tRNA-Met | 14512 | 14580 | 69 |  |  | 0 | + |
| ND2 | 14581 | 15579 | 999 | ATT | TAA | 4 | + |
| tRNA-Trp | 15584 | 15652 | 69 |  |  | -1 | + |
| tRNA-Cys | 15652 | 15718 | 67 |  |  | -1 | - |
| tRNA-Tyr | 15718 | 15785 | 67 |  |  | 1 | - |

* TAA stop codon is completed by the addition of 3' A

**References**

Crandall, K. A., & Fitzpatrick, J. F. (1996). Crayfish Molecular Systematics: Using a Combination of Procedures to Estimate Phylogeny. *Systematic Biology*, *45*(1), 1–26. <https://doi.org/10.2307/2413509>

Folmer, O., Black, M., Hoeh, W., Lutz, R., & Vrijenhoek, R. (1994). DNA primers for amplification of mitochondrial cytochrome c oxidase subunit I from diverse metazoan invertebrates. *Molecular marine biology and biotechnology*, *3*(5), 294–299.

Machida, R. J., Miya, M. U., Nishida, M., & Nishida, S. (2002). Complete mitochondrial DNA sequence of Tigriopus japonicus (Crustacea: Copepoda). *Marine biotechnology (New York, N.Y.)*, *4*(4), 406–417. <https://doi.org/10.1007/s10126-002-0033-x>

Miller, A. D., & Austin, C. M. (2006). The complete mitochondrial genome of the mantid shrimp Harpiosquilla harpax, and a phylogenetic investigation of the Decapoda using mitochondrial sequences. *Molecular phylogenetics and evolution*, *38*(3), 565–574. <https://doi.org/10.1016/j.ympev.2005.10.001>

Mondal, D & Mandal, N. (2020). Molecular phylogeny of mitochondrial DNA: Shrimp species identification by multiplex and real-time PCR. *Food Control*. 108, 106868. <https://doi.org/10.1016/j.foodcont.2019.106868>

Kitaura, J., Wada, K., & Nishida, M. (1998). Molecular phylogeny and evolution of unique mud-using territorial behavior in ocypodid crabs (Crustacea: Brachyura: Ocypodidae). *Molecular biology and evolution*, *15*(6), 626–637. <https://doi.org/10.1093/oxfordjournals.molbev.a025966>

Viker, S. M., Klingberg, Å. N., & Sundberg, P. (2006). The complete mitochondrial DNA sequence of the northern shrimp, Pandalus borealis. *Journal of Crustacean biology*, *26*(3), 433-435. <http://www.jstor.org/stable/4094127>

Yamauchi, M. M., Miya, M. U., & Nishida, M. (2002). Complete mitochondrial DNA sequence of the Japanese spiny lobster, Panulirus japonicus (Crustacea: Decapoda). *Gene*, *295*(1), 89-96. <https://doi.org/10.1016/S0378-1119(02)00824-7>

Yamauchi, M. M., Miya, M. U., Machida, R. J., & Nishida, M. (2004). PCR-based approach for sequencing mitochondrial genomes of decapod crustaceans, with a practical example from kuruma prawn (Marsupenaeus japonicus). *Marine biotechnology (New York, N.Y.)*, *6*(5), 419–429. <https://doi.org/10.1007/s10126-003-0036-2>
